# Hypothyroidism Manifesting as a Combination of Ascites and Malnutrition Requiring Total Parenteral Nutrition: A Unique Presentation

**DOI:** 10.7759/cureus.5338

**Published:** 2019-08-07

**Authors:** Yousaf Zafar, Stephanie R Suddaby, Muhammad Shafiq

**Affiliations:** 1 Internal Medicine, University of Missouri-Kansas City School of Medicine, Kansas City, USA; 2 Internal Medicine, University of Missouri - Kansas City School of Medicine, Kansas City, USA; 3 Internal Medicine, The University of Kansas Medical Center, Kansas City, USA

**Keywords:** hypothyroidism, ascites, severe nutrition

## Abstract

Ascites is the abnormal buildup of fluid in the abdomen. Despite appropriate workup, including diagnostic paracentesis with fluid analysis and abdominal imaging, the cause of ascites is sometimes unknown. In this case, testing for less common causes should be performed, including checking thyroid function test (TFT) because hypothyroidism has also been reported to be a rare cause of ascites. Patients may also have concomitant malnutrition as an effect of severe hypothyroidism, rather than as its cause. We report the case of a 62-year-old female with a history of hypothyroidism and non-compliance who presented with unexplained ascites and then also developed severe malnutrition, requiring total parenteral nutrition (TPN). Extensive testing, including laparotomy, was unable to reveal the cause of ascites and malnutrition until the patient mentioned, during her hospital stay, non-compliance with her home dose of levothyroxine (175 μg) because of the cost. TFT results indicated that the patient had severe hypothyroidism, with a thyroid-stimulating hormone (TSH) level of 21.9 IU/mL and a free thyroxine level (T4) level of 0.2 IU/mL. The patient’s home levothyroxine dose was resumed. The patient clinically improved and was discharged on an oral diet. The patient’s ascites resolved completely, the TSH level was 2.39 IU/mL, and the T4 level was 1.7 IU/mL at the eight-week follow-up.

## Introduction

Ascites refers to accumulated fluid within the peritoneal cavity. The pathophysiology of ascites includes portal hypertension [1], hypoalbuminemia [2], and/or peritoneal disease [3], which then dictates the nature of ascites as either exudative or transudative. Less common causes of ascites include pancreatic ascites (which results from local inflammation in acute pancreatitis) [4], injury to the genitourinary tract [5], and, occasionally, hemoperitoneum.

The development of ascites is pathologic and, therefore, diagnostic paracentesis should be performed for every new onset of ascites to determine the cause [6]. The serum ascites albumin gradient (SAAG), which is obtained through diagnostic paracentesis, helps classify the ascites as either the result of portal hypertension (if SAAG >1.1) or not (if SAAG is <1.1). SAAG <1.1 mostly occurs in exudative ascites, with the important exception of nephrotic syndrome. Exudative effusion commonly results from peritoneal disease. This helps narrow down the potential cause based on the pathophysiology (as stated above). Additionally, other ascitic fluid analyses (such as ascitic fluid cell count, cultures, and cytology) and abdominal imaging are used together to find the definitive cause, which can be subsequently treated.

Despite identifying the fluid as either exudative/transudative or calculating the SAAG value, the exact cause of ascites may remain elusive. We present a case where the patient was found to have SAAG >1.1 on diagnostic paracentesis but further basic ascitic fluid analysis and abdominal imaging did not reveal the exact cause. The ascites was also accompanied by malnutrition, requiring total parenteral nutrition (TPN). This is a rare occurrence, where serum chemistry led to the final diagnosis and treatment of both ascites and the accompanying malnutrition.

## Case presentation

A 62-year-old female with a history of hypothyroidism (diagnosed 26 years ago) and non-compliance presented to the emergency department (ED) with increasing abdominal girth for the past two months, which was associated with decreased appetite, unintentional weight loss (30 pounds over the same time), nausea, and diarrhea. She denied any alcohol consumption or history of hepatitis B or C viral infection.

In the ED, the patient had a temperature of 98.2°F, blood pressure of 131/87 mmHg, heart rate of 110/minute, respiratory rate of 16 per minute, and was maintaining an oxygen saturation of 99% on pulse oximetry on room air. Positive physical exam findings included tachycardia without any murmurs or rhythm abnormalities, 1+ pitting edema in the bilateral lower extremities, and markedly distended abdomen with a positive fluid wave.

Complete blood count revealed hemoglobin of 11.3 g/dL with microcytosis (mean corpuscular volume of 69 fL), white blood cell (WBC) count of 3.6 × 103/µL, with normal differentials and a platelet count of 369 × 103/µL. Complete metabolic panel results were unremarkable except for a mildly elevated aspartate aminotransferase level of 47 U/L (upper limit of normal, 40 U/L) and hypokalemia with a potassium level of 2.9 mEq/L. The international normalized ratio was 1.0 and the acute viral hepatitis panel results for hepatitis A, B, and C were also unremarkable. The patient subsequently underwent a computed tomography (CT) scan of the abdomen/pelvis with contrast, which demonstrated marked small bowel dilation with mesenteric swirling in her left-mid abdomen along with a large amount of ascites.

**Figure 1:**
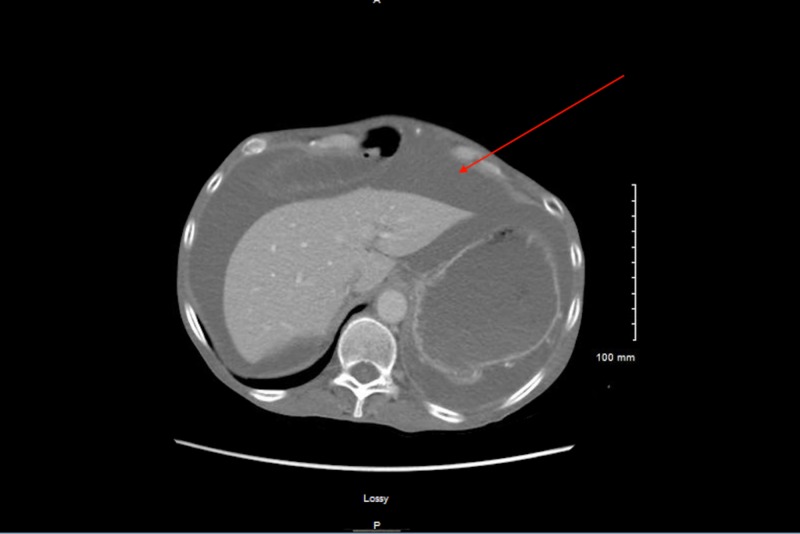
Computed tomography showing ascites

The patient had a nasogastric (NG) tube placed because there was a concern of small bowel obstruction. However, despite the NG tube placement, her distension worsened. The general surgery team was consulted based on the CT scan findings but they felt that the patient’s symptoms were more chronic in nature and the patient would have been sicker if she had a true acute small bowel obstruction that needed surgical intervention. The gastroenterology (GI) team was also consulted and the patient underwent both diagnostic and therapeutic paracentesis with 2370 mL fluid removal. The cell count analysis of the ascitic fluid was unremarkable and cytology was also negative for malignancy. The ascitic fluid analysis revealed a SAAG of 2.4 (i.e. which was >1.1) and a total ascitic fluid protein level of 3.1 g/dL (which was >2.5 g/dL). These results initially prompted the thought that the patient could have cardiac ascites. Therefore, the patient underwent a transthoracic echocardiogram, which showed normal left ventricular systolic function and an ejection fraction of 60% with no significant valvular abnormalities. The CT scan abdomen and abdominal ultrasound (which was also performed) did not suggest any carcinomatosis or adnexal mass. Both also showed that the patient’s liver was of normal size and had a normal appearance (Figure 1).

Because there was no certain cause of ascites and persistent demonstration of distension with concern for small bowel obstruction, the patient eventually underwent exploratory laparotomy. It demonstrated abdominal ascites, a single intra-abdominal gelatinous mass, and a diffusely dilated small and large bowel without necrosis. Ascitic fluid sampled from the exploratory laparotomy and the gelatinous mass pathology were both negative for the malignant process.

During this workup, the patient was unable to maintain nutrition at goal. Therefore, TPN had to be initiated for severe malnutrition. At this point, the GI team began to investigate less common causes of ascites and ordered serologic testing (the results of which were within normal limits), including immunoglobulin (Ig) A (IgA) (348 mg/dL), IgM (80 mg/dL), IgG (1158 mg/dL), ceruloplasmin (27.0 mg/dL), fibrinogen (351 mg/dL), total cholesterol (88 mg/dL), and transferrin (200 mg/dL). Alpha 1 antitrypsin was also negative. Push enteroscopy was also performed, and it demonstrated a normal esophagus, duodenum, and jejunum. The patient’s stomach had erythema in the antrum, which was compatible with gastritis only. Small bowel biopsies were taken and demonstrated normal mucosa.

The patient was supposed to be taking levothyroxine 175 μg orally daily. Later during her hospital stay, the patient admitted that she had not been taking her thyroid medications for many months because they were too expensive. TFTs were ordered and the results showed a TSH level of 21.9 IU/mL and a T4 level of 0.2 IU/mL. Endocrinology was consulted because of concerns that severe hypothyroidism was causing the patient’s symptoms, including ascites and malnutrition, because the results of all other tests (as outlined above) were negative for ascites.

The patient was started on 175 μg of levothyroxine while in the hospital, and she clinically improved. Her abdominal pain resolved and she was no longer complaining of diarrhea. TPN was discontinued and the patient tolerated food by mouth. A repeat CT scan of the abdomen/pelvis performed at eight weeks after discharge noted only a small quantity of residual ascites, and at this follow-up with her primary care physician, she noted much improvement in her symptoms. Follow-up thyroid studies at eight weeks showed a TSH level of 2.39 IU/mL and a T4 level of 1.7 IU/mL.

## Discussion

One of the first cases of ascites resulting from hypothyroidism was reported in 1950 in the New England Journal of Medicine [7]. Although it is rare for ascites to develop in patients with hypothyroidism, it has been reported [8-12]. Watanakunakorn et al., in their study of 400 myxedema patients, reported that 15 patients also had ascites [13]. Our case is unique because our patient had ascites, which is rare, and developed malnutrition to the point that TPN was required.

Malnutrition can lead to hypothyroidism, which is common in certain developing and under-developed countries [14]. However, the authors could not find any reasonable article that would report hypothyroidism as the cause of severe malnutrition. A study by Tahara et al. described a 68-year-old male, without any previous thyroid disease, who developed transient primary hypothyroidism that was associated with protein-calorie malnutrition (PCM) [15]. In this patient, the researchers investigated the change of thyroid function during protein-calorie repletion using TPN. Iodine was removed from the nutrients and TPN with full amino acid supplementation was administered to rule out iodine deficiency as the cause of the primary hypothyroidism this case. Despite the removal of iodine, serum T4, and triiodothyronine (T3) suddenly increased from 1.1 μg/dL and <25 ng/dL to 3.5 μg/dL and 59 ng/dL, respectively. Serum TSH decreased from 120 μU/mL to 17 μU/mL in a few days after starting TPN and reached a level that was within the normal range in four weeks. These results showed that the T4 synthesis was extremely impaired by PCM despite strong stimulation by TSH, and suppression of T4 synthesis by PCM led the patient to recurrently be in primary hypothyroidism [15]. However, our patient’s ascites did not improve with TPN, making malnutrition less likely to be the cause of uncontrolled hypothyroidism but more likely an effect of it.

Diagnostic paracentesis is recommended for every new-onset ascites and subsequent ascitic fluid analysis to help identify the potential cause [6]. The protein content of the ascitic fluid associated with hypothyroidism is mostly elevated. However, the SAAG value can be high or low. There are multiple theories to explain ascites in hypothyroidism such as a decrease in free water clearance by the kidneys because of excessive antidiuretic hormone in hypothyroidism or an increase in capillary permeability because of low T4 levels. However, the exact cause of ascites in hypothyroidism remains unknown. Similarly, it was not clear how hypothyroidism led to severe malnutrition in our patient.

The malnutrition and ascites in our patient did not improve until her correct home dose of levothyroxine was started during her hospital stay. Only after resuming her thyroid treatment did the patient’s ascites resolve and her appetite improve along with her nutritional status. The patient was on an oral diet at the time of discharge from the hospital.

## Conclusions

Severe hypothyroidism can be the cause of unexplained ascites and, therefore, TFTs should be ordered if the etiology of ascites remains unclear. This case report highlights that malnutrition can also result from hypothyroidism. Therefore, if TFTs do not change after TPN is initiated, T4 replacement should be started because hypothyroidism is likely the cause of malnutrition, not an effect of it.
